# Taxonomically and Functionally Distinct Ciliophora Assemblages Inhabiting Baltic Sea Ice

**DOI:** 10.1007/s00248-021-01915-4

**Published:** 2021-11-08

**Authors:** Markus Majaneva, Janne-Markus Rintala, Jaanika Blomster

**Affiliations:** 1 Norwegian Institute for Nature Research (NINA), Trondheim, Norway; 2 Tvärminne Zoological Station, University of Helsinki, Hanko, Finland; 3 Ecosystems and Environment Research Programme, Faculty of Biological and Environmental Sciences, University of Helsinki, Helsinki, Finland; 4 Institute for Atmospheric and Earth System Research, Faculty of Agriculture and Forestry (INAR), University of Helsinki, Helsinki, Finland

**Keywords:** DNA metabarcoding, Phylogenetic placement, Mixotrophy, Predator–prey interactions, Winter ecology

## Abstract

Ciliophora is a phylum of unicellular eukaryotes that are common and have pivotal roles in aquatic environments. Sea ice is a marine habitat, which is composed of a matrix of solid ice and pockets of saline water in which Ciliophora thrive. Here, we used phylogenetic placement to identify Ciliophora 18S ribosomal RNA reads obtained from wintertime water and sea ice, and assigned functions to the reads based on this taxonomic information. Based on our results, sea-ice Ciliophora assemblages are poorer in taxonomic and functional richness than under-ice water and water-column assemblages. Ciliophora diversity stayed stable throughout the ice-covered season both in sea ice and in water, although the assemblages changed during the course of our sampling. Under-ice water and the water column were distinctly predominated by planktonic orders Choreotrichida and Oligotrichida, which led to significantly lower taxonomic and functional evenness in water than in sea ice. In addition to planktonic Ciliophora, assemblages in sea ice included a set of moderately abundant surface-oriented species. Omnivory (feeding on bacteria and unicellular eukaryotes) was the most common feeding type but was not as predominant in sea ice as in water. Sea ice included cytotrophic (feeding on unicellular eukaryotes), bacterivorous and parasitic Ciliophora in addition to the predominant omnivorous Ciliophora. Potentially mixotrophic Ciliophora predominated the water column and heterotrophic Ciliophora sea ice. Our results highlight sea ice as an environment that creates a set of variable habitats, which may be threatened by the diminishing extent of sea ice due to changing climate.

**Supplementary Information:**

The online version contains supplementary material available at 10.1007/s00248-021-01915-4.

## Introduction

Members of the phylum Ciliophora are ubiquitous and diverse unicellular eukaryotes [1, 2]. They are an important trophic link. They feed on bacteria and photosynthetic unicellular eukaryotes, predate other unicellular eukaryotes, parasitize a wide range of organisms, and are an important energy resource for higher-level components of diverse trophic networks [3, 4]. Although heterotrophic, many Ciliophora have acquired phototrophy. Those Ciliophora catch and harbour phototrophic symbionts or sequester plastids from the captured photosynthetic unicellular eukaryotes. They are therefore functionally not solely consumers but exhibit different states of the mixotrophic continuum [5]. Therefore, understanding the diversity and functions of Ciliophora in a habitat is essential to fully perceive any ecological processes, for example dynamics of trophic networks and carbon cycling [6].

Sea ice covers 3–6% of the earth’s surface annually [7]. It is a heterogeneous semi-solid matrix in which solid ice alternates with saline water in brine channels and pockets. The diameter of the brine formations is from micrometres to several centimetres [8], and the geometry and volume correlate with the salinity of the parent water and temperature. For example, in the brackish Baltic Sea (surface salinity 1–9), the sea-ice brine channels are from micrometres to millimetres in diameter and are of a relatively low volume [9]. Nevertheless, the brine channels are inhabited by an active community of photosynthetic and heterotrophic organisms [9–11]. Due to the small size of the brine channels, the largest organisms within the Baltic Sea ice are scarcely encountered rotifers and copepod nauplii [9–11]. Sea-ice organisms prefer capillaries that are only slightly larger than themselves, and microorganisms can cover 6–41% of the brine surface area [12], which is high compared with soils where less than 1% of the surface area is covered by organisms [13]. The sea-ice organisms may also restructure the channels to be more complex and habitable using extracellular polymeric substances [14]. Thus, the narrow brine channels of the Baltic Sea ice provide effective refuges from zooplankton and fish predation and food-dense habitats for unicellular eukaryotes, such as Ciliophora. The Baltic Sea ice is an ideal habitat to study the diversity and ecological role of ice-associated Ciliophora.

The phylum of Ciliophora is divided into 11 classes [1]. Classes Spirotrichea, Phyllopharyngea and Oligohymenophorea include over two-thirds of the described species, and these classes are also well represented in sea ice [10, 15–19]. Generally, the sea-ice Ciliophora community is dominated by Oligotrichida (an order within Spirotrichea), and their abundance has a clear seasonality, which follows primary and bacterial production: it is low during the dark period, but towards the spring with increasing light as well as photosynthetic growth and bacterial production, Ciliophora abundance gets higher [10, 20–22]. Also, species from the classes Litostomatea, Armophorea, Heterotrichea, Prostomatea, Nassophorea and Colpodea have been found to live in the sea ice [16, 19, 23, 24]. Members of the classes Karyorelictea and Plagiopylea, which are typical for anaerobic habitats, seem to be absent from sea ice [16].

Reliable species-level morphological identification of Ciliophora requires laborious live observation and staining techniques [25]. However, surveys based on sequencing of the 18S ribosomal RNA (18S rRNA) gene have become widely used during the last two decades because of the much higher throughput of the DNA-based method compared with the microscopy-based method. The DNA-based method has several limitations, including gene copy and primer binding efficiency differences and sequencing errors that can be mitigated computationally to some extent [26, 27]. Further, DNA-based identification relies heavily on quality and a sufficient amount of reference sequences: the more comprehensive and better annotated the reference library, the more accurate the possible identifications [28]. A recent annotation effort generated the most comprehensive reference library of annotated Ciliophora sequences to date with an 18S rRNA gene reference tree, which substantially improves classification of Ciliophora in DNA-based analyses [29].

As for morphological identification of Ciliophora, depicting their feeding behaviour and trophic modes requires laborious experiments and microscopic observations. A respectable amount of this work has been performed on Ciliophora and reviewed in, for example, [1, 30, 31]. Adl et al. [2] compiled trophic functional groups across unicellular eukaryote diversity, and argue that it is safe to assume that in most cases species within a genus are most likely to have the same trophic function. However, mixotrophic Ciliophora can be tricky in this sense. The genus *Mesodinium* is an excellent example—the genus includes *Mesodinium rubrum*, which is almost exclusively phototrophic [32], as well as not as strictly phototrophic *Mesodinium chamaeleon* and heterotrophic *Mesodinium pulex* [33]. The same holds for the genus *Strombidium*, of which many have acquired phototrophy [34]. Therefore, care must be taken when assigning functions to Ciliophora identified based on 18S rRNA genes and interpreting the results. Here, we assigned taxa as potentially mixotrophic to take this uncertainty into account.

In this study, we firstly placed the Ciliophora sequences we had gathered during our sea-ice studies in the Baltic Sea [11, 35–37] on the Ciliophora 18S rRNA reference tree [29]. With this reidentification, we gained more accurate taxonomic information on the sea-ice associated Ciliophora assemblages and could assign functions to them. Secondly, using a time series [36, 37], we tested three hypotheses. These hypotheses (H) were: (H1) sea ice, under-ice water (water immediately under the ice cover) and the water column (deeper water under the ice cover) have the same taxonomic and functional Ciliophora richness and evenness; (H2) sea ice, under-ice water and the water column have the same taxonomic and functional Ciliophora composition; (H3) Ciliophora diversity, assemblages and functions stay the same throughout the ice-covered season.

## Methods

The presented sequence material (reads) is from Baltic Sea ice studies based on sequencing of the 18S rRNA gene [11, 35–37] (see Fig. S1 and Table S1 (Online Resource 1) for sampling locations and time points). Earlier studies [11, 35] concentrated on sea ice, while the time series [36, 37] included a similar number and amount of sea-ice, under-ice water and water-column samples (2 L per sample [36]), and therefore only the time-series data are used for hypothesis testing. The Sanger-sequenced reads are available in the European Molecular Biology Laboratory Nucleotide Sequence Database (FN689869–FN690738 [35]). The 454 GS FLX Titanium (Hoffmann-La Roche, Basel, Switzerland) generated raw reads are available at the Sequence Read Archive of the European Nucleotide Archive (ENA SRA) with accession number PRJEB7625 [11]. The Illumina (San Diego, CA, USA) MiSeq v3 600-cycle kit raw reads are available at the ENA SRA repository with accession numbers PRJEB21047 [36] and PRJEB25089 [37]. All reads affiliated with Ciliophora in these studies were selected for subsequent analyses in this study (see Supplementary methods (Online Resource 1) for details).

Our Sanger-sequenced Ciliophora reads were 650–1500 bp long and covered positions 30–1710 of the complete *Tetrahymena farleyi* 18S rRNA gene (accession number AF184665.1). Our 454 sequenced reads were 339–453 bp long, covering positions 1264–1710 of the *T. farleyi* 18S gene (including the variable regions V7–V9), and our Illumina MiSeq reads were 294–532 bp long, covering positions 583–1104 of the *T. farleyi* 18S gene (including the variable region V4). All our short reads that were identical to a longer one were merged with the longer ones. The Ciliophora 18S rRNA gene reference sequences and tree [29] were downloaded on 29 March 2019. Our reads were aligned together with the reference sequences using the MAFFT online service [38], and the resulting alignment was cut to the length of our longest Sanger reads (alignment length 5297 bp). The aligned reads were placed on the reference tree using the evolutionary placement algorithm EPA-ng v0.3.5 [39]. The placements were visualized using the Interactive Tree Of Life v3 [40]. In addition, our reads were classified with the assign_taxonomy command of DADA2 [27] using the EukRef annotated Ciliophora sequences [29] and the PR^2^ database [41] as references. The lowest taxonomic level with bootstrap support over 80% was accepted. We used phylogenetic placement for taxonomic identification, but present also the DADA2 classified results in Table S2 (Online Resource 2). Since many reads were still assigned to a family or higher taxonomic level, the reads were clustered into 98% operational taxonomic units (OTUs) using mothur 1.42.3 [42], representing probable different species within those higher-level taxa (220 OTUs). The ecological role (feeding type categories according to Adl et al. [2], living mainly on surfaces or as plankton, heterotrophy or potential mixotrophy) of each taxon was based on Lynn [1], Adl et al. [2] and Stoecker and Lavrentyev [43] but refined if more detailed information was available (literature cited in Table S2 in Online Resource 2).

For statistical analyses, Ciliophora richness was calculated as the number of OTUs present in samples (normalized to 39,861 reads/sample) and Ciliophora evenness as e^H^/S [44], where H is the Shannon index [45] and S is the number of taxa. Both diversity measures were compared among different sample types with one-way analysis of variance (ANOVA) and following Tukey’s pairwise comparisons. Non-metric multidimensional scaling (NMDS) as well as redundancy and variance partitioning analyses were run using the R package vegan [46] with scripts mod(), capscale() and varpart(). The process of neutral drift is likely to cause a trend in the data series [47]. To separate this drift from trends induced by environmental or biotic processes in the time series [36], asymmetric eigenvector maps (AEMs) and local contributions to beta diversity (LCBD) were generated, following Appendix S2 of Legendre and Gauthier [48]. Values of sea-ice and water temperature, salinity, nutrients, algal biomass and chlorophyll *a* were taken from Enberg et al. [36] and values of bacterial abundance and productivity from Kaikkonen et al. [49]. Irradiance and air temperature were retrieved from the Photovoltaic Geographical Information System (https://ec.europa.eu/jrc/en/pvgis) 21 September 2020, and a weekly average (7-day period prior to the sampling time) of the daily irradiance and air temperature was calculated for each sampling time. The details of the steps are presented in Online Resource 3.

## Results

### Taxonomic and Functional Composition of the Ciliophora Assemblages

**Fig. 1:**
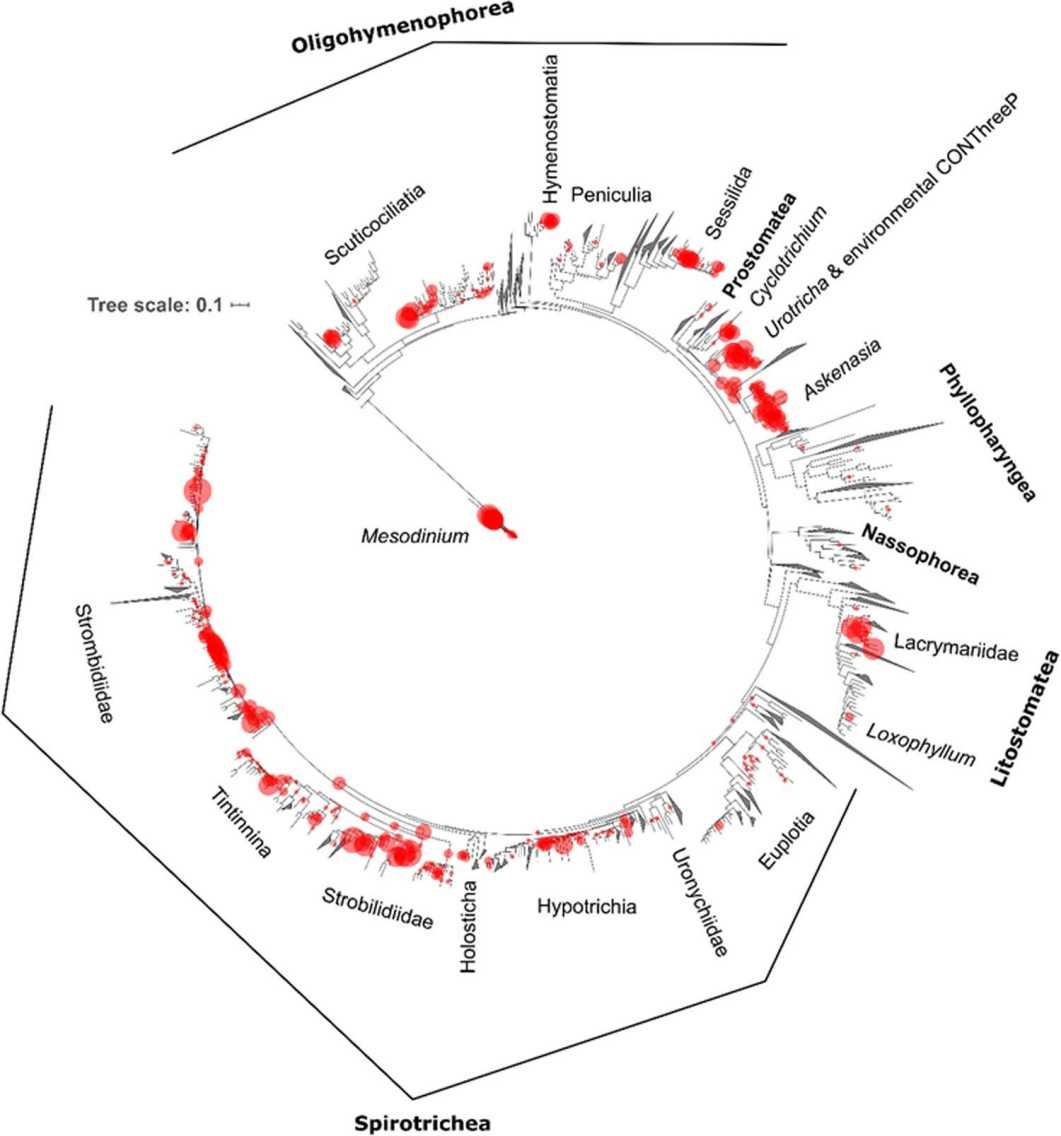
Baltic Sea ice associated Ciliophora placed in an unrooted 18S ribosomal RNA gene reference tree [29]. The clades of the reference tree that do not contain Baltic Sea ice associated Ciliophora are collapsed. The classes that include Baltic reads are written in bold. The size of the circle is proportional to the number of unique reads placed at the given node

We obtained 480,000 Ciliophora reads (508 unique reads, 220 different 98% OTUs) from our 74 wintertime ice and water samples. The richest class of Ciliophora was Spirotrichea, which contained 106 OTUs (Table S2 in Online Resource 2), followed by Prostomatea and environmental CONThreeP clades, which were closely affiliated with Prostomatea (1 and 48 OTUs, respectively; Fig. 1). The CONThreeP clade is a supercluster within the subphylum Intramacronucleata comprising the classes Colpodea, Oligohymenophorea, Nassophorea, Plagiopylea, Prostomatea and Phyllopharyngea [2]. The next richest classes were Oligohymenophorea (40 OTUs) and Litostomatea (9 OTUs). Seven OTUs were affiliated with *Mesodinium* (uncertain position within the SAL supercluster comprising the classes Spirotrichea, Armophorea and Litostomatea within the subphylum Intramacronucleata). In addition, we found 4 Phyllopharyngea OTUs, 2 Nassophorea OTUs and 3 OTUs that could not be affiliated with any of the classes (two classified within the SAL supercluster and one within the subphylum Intramacronucleata).

The Choreotrichida were the richest Spirotrichea order with 49 OTUs (26 OTUs within the family Strobilidiidae, 9 within the family Tintinnina, and 14 other Choreotrichida), followed by the order Oligotrichida with 35 OTUs (33 within the family Strombidiidae and 2 within the family Tontoniidae). Most of the environmental CONThreeP OTUs were affiliated with the genus *Askenasia* (26 OTUs), with the unassigned environmental CONThreePs (12 OTUs) and with the genus *Urotricha* (7 OTUs). Oligohymenophorea OTUs were mostly affiliated with the subclass Scuticociliatia (21 OTUs) and the subclass Peritrichia (10 OTUs). Most of the Litostomatea OTUs were affiliated with the family Lacrymariidae (5 OTUs).

Our reads were divided into 11 different feeding types (Table S2 in Online Resource 2). Omnivory (feeding on both bacteria and unicellular eukaryotes) was distinctly the most common feeding type: 164 OTUs were categorized as omnivorous and 3 OTUs as omnivorous/cytotrophic (cytotrophic is feeding on unicellular eukaryotes [2]). The omnivores were also the most abundant Ciliophora as 95.7% of the Ciliophora reads were categorized as omnivorous and 0.01% as omnivorous/cytotrophic. Bacterivorous Ciliophora were the second richest feeding type with 16 OTUs (1.5% of the reads), followed by cytotrophic (8 OTUs and 0.2% of the reads), cytotrophic/detritivorous (8 OTUs, 1.7%), predatory (8 OTUs, 0.4%) and parasitic (7 OTUs, 0.4%) Ciliophora. Three OTUs were categorized as bacterivorous/histophagous (0.01%), one as a commensal bacterivore and one feeding on algal filaments (a few reads of both). More OTUs were categorized as planktonic (181 OTUs, 97.5% of the reads) than as surface oriented (39 OTUs, 2.5%). While more OTUs (142) were categorized as heterotrophic than as potentially mixotrophic (78 OTUs), the read abundance of the potentially mixotrophic Ciliophora (53.7% of the reads) was higher than that of the heterotrophic ones (46.3%).

### H1—Diversity Measures Differ in Sea Ice and Water Habitats

**Fig. 2:**
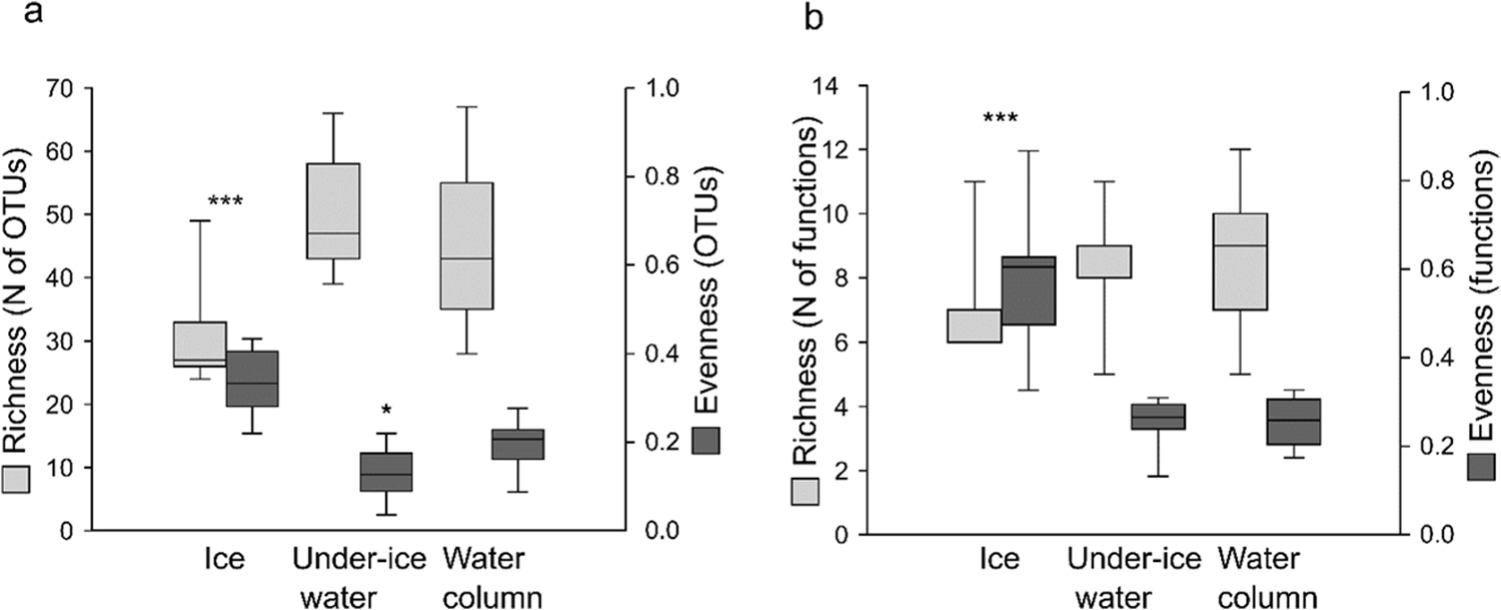
Number (*N*) and evenness of 98% Ciliophora operational taxonomic units (OTUs) and functions in the time-series samples, based on normalized number of reads (39,861 reads/sample). (**a**) Sea-ice samples (***, 15 samples) had significantly lower richness but significantly higher evenness than under-ice water (15 samples) and water-column samples (16 samples) according to one-way analysis of variance (ANOVA) (richness: *F* = 22.09, *p* = 2.70E-6; evenness: *F* = 54.26, *p* = 1.74E-12) and following Tukey’s pairwise comparisons (richness: ice–under-ice water Tukey’s *Q* = 8.18, *p* = 0.00013; ice–water column: *Q* = 5.93, *p* = 0.00050; evenness: ice–under-ice water Tukey’s *Q* = 14.59, *p* = 0.0001; ice–water column: *Q* = 9.82, *p* = 0.0001). In addition, under-ice water samples (*) had significantly lower evenness than water-column samples (Tukey’s *Q* = 4.77, *p* = 0.005). (**b**) Sea-ice samples (***) had significantly fewer but significantly more evenly distributed functions than under-ice water and water-column samples according to one-way ANOVA (richness: *F* = 4.94, *p* = 0.012; evenness: *F* = 67.68, *p* = 5.21E-14) and following Tukey’s pairwise comparisons (richness: ice–under-ice water: Tukey’s *Q* = 3.74, *p* = 0.03; ice–water column: *Q* = 3.98, *p* = 0.02; evenness: ice–under-ice water: Tukey’s *Q* = 14.30, *p* = 0.0001; ice–water column: *Q* = 14.34, *p* = 0.0001)

Sea-ice samples (*N* = 15) had significantly lower Ciliophora taxon richness and fewer functions than under-ice water (*N* = 15) and water-column samples (*N* = 16) in the time series (Fig. 2). In addition, Ciliophora read abundance was significantly lower in sea-ice samples than under-ice water and water-column samples despite significantly higher total read abundance in sea-ice samples than under-ice water and water-column samples (Fig. S2 in Online Resource 1). In contrast, both taxon evenness and function evenness were significantly higher in sea ice than in under-ice water and water-column samples. Taxon evenness was also higher in water-column than in under-ice water samples.

### H2—Different Ciliophora Assemblages in Sea Ice, Under-Ice Water and the Water Column

**Fig. 3:**
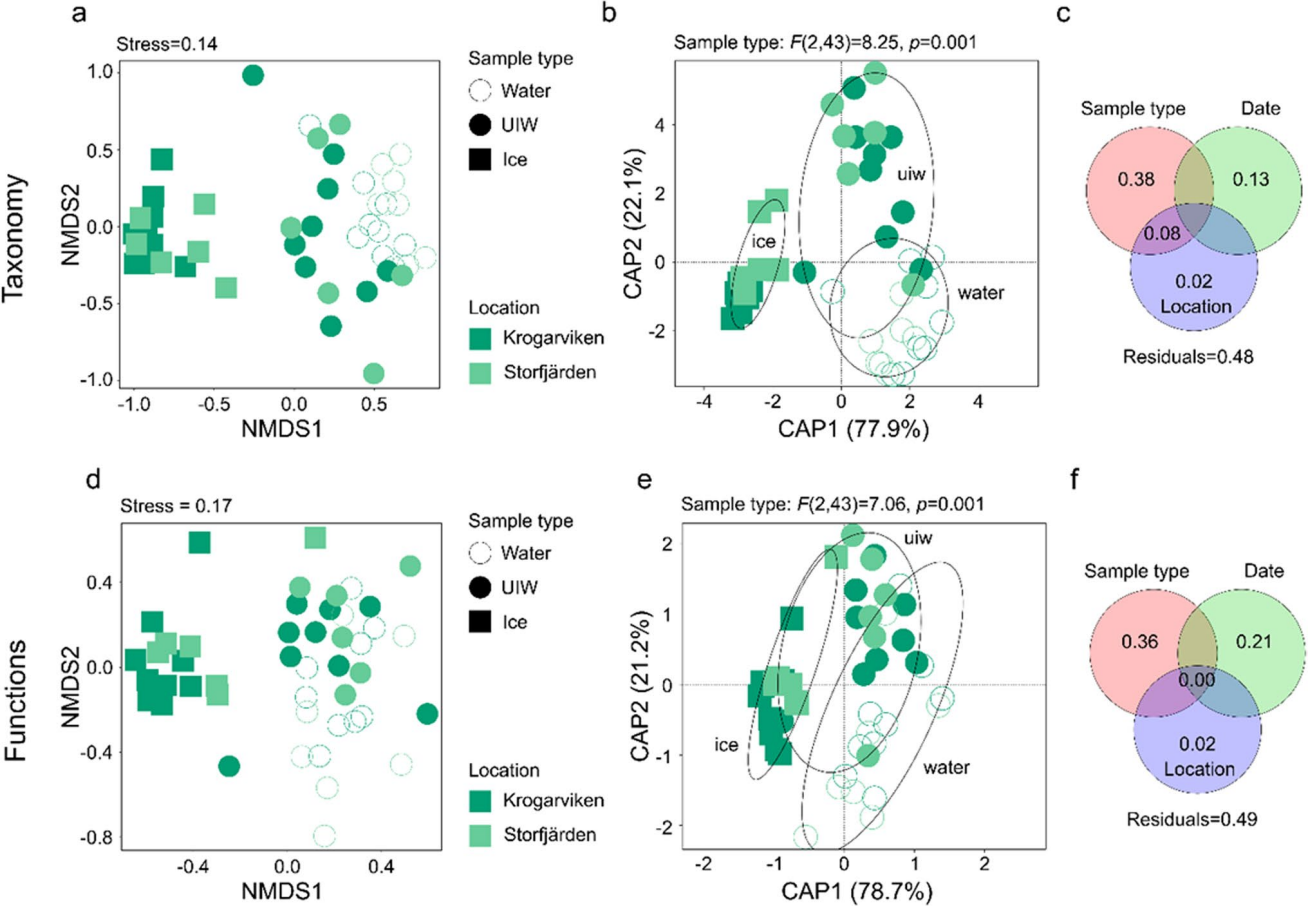
Differences in Ciliophora assemblages and their functions. (**a**, **d**) Non-metric multidimensional scaling (NMDS) plots based on Bray–Curtis dissimilarity indices of the Ciliophora assemblages in the different samples. (**a**) Taxonomic composition, (**d**) functional composition. (**b**, **e**) Distance-based redundancy analysis plots based on binomial distribution of the Ciliophora assemblages in the different samples. Significance was tested with a permutation test (999 permutations, significance level *p* < 0.05) and following pairwise Adonis with Holm-corrected *p*-values. (**b**) Taxonomic composition. All sample types differed significantly (*p* = 0.003). (**e**) Functional composition. Functions in ice differed significantly from functions in water and under-ice water (UIW, *p* = 0.003) and functions in UIW differed significantly from functions in the water column (*p* = 0.015). (**c**, **f**) Venn diagrams showing variation partitioning for Ciliophora assemblages

**Fig. 4:**
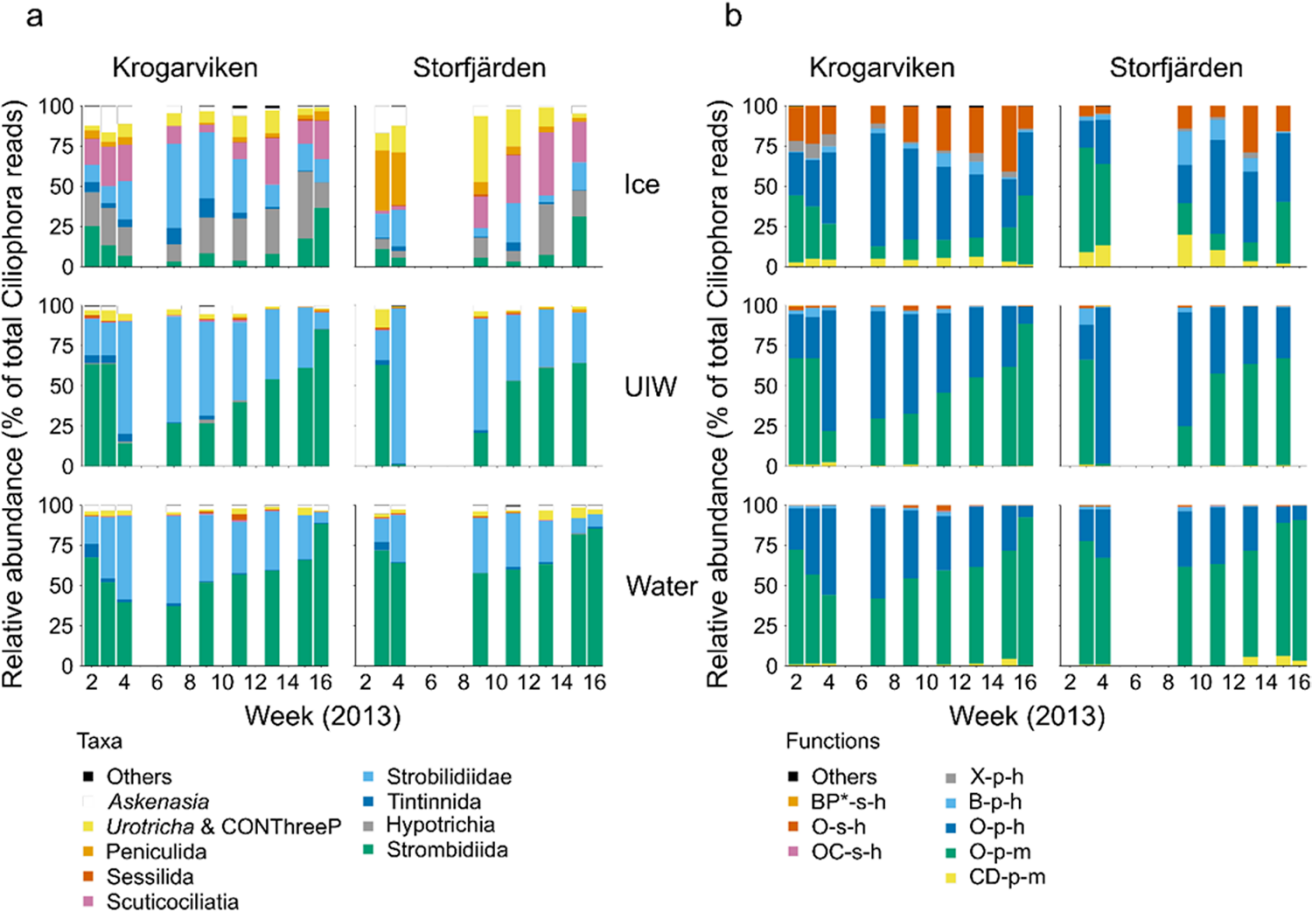
Ciliophora succession. (**a**) Relative abundance of 98% Ciliophora operational taxonomic units (OTUs) in sea ice, under-ice water (UIW) and the water column at two time-series sampling stations. (**b**) Relative abundance of Ciliophora functional groups in sea ice, under-ice water and the water column at two time-series sampling stations. The abbreviated functions include: (B) bacteria filtration, (P*) histophagous, (O) omnivorous, (C) cytotrophic, (X) parasitic, (D) occasionally detritivorous, (s) surface dwelling, (p) planktonic, (h) heterotrophic and (m) potentially mixotrophic. OTUs and functions were merged to show the most abundant groupings

The different sample types grouped significantly apart, i.e. the Ciliophora assemblage within sea ice, differed from that in the under-ice water and water column (Fig. 3a, b). The highest variability was due to sample type (38% of variation was explained solely by sample type, Fig. 3c). Strobilidiidae (Choreotrichida) and Strombidiidae (Oligotrichida) predominated in under-ice water and water-column samples (Fig. 4a). Strobilidiidae was the most abundant group in under-ice water—on average 49.9% of the under-ice water reads were Strobilidiidae. In the water column, Strombidiidae was the predominant group (62.6% of the reads). Although the read abundances of Strobilidiidae and Strombidiidae were different, the same Strobilidiidae (Strobilidiidae sp. 4) and Strombidiidae (Strombidiida sp. 1, Strombidiida sp. 3 and *Strombidium paracapitatum* 1) OTUs had the highest read abundances in under-ice water and water-column samples (Table S2 in Online Resource 2).

The read abundance of the different Ciliophora groups was more evenly distributed in sea ice than in under-ice water or the water column (Fig. 4a). Strobilidiidae reads were the most abundant (23.7%) in sea ice, followed by Scuticociliatia (16.6%), subclass Hypotrichia (15.8%), Strombidiidae (12.1%), *Urotricha* and unassigned environmental CONThreeP (11.4%), order Peniculida (8.8%) and *Askenasia* (7.7%) reads. Furthermore, the Strobilidiidae OTUs with the highest read abundances were not the same in sea-ice samples and in under-ice water or water-column samples. In sea ice, Strobilidiidae sp. 1 and *Rimostrombidium* sp. 1 OTUs had the highest read abundances of the Strobilidiidae (Table S2 in Online Resource 2). The same difference was evident in other groups as well. For example, *Askenasia* sp. 1 was predominant in sea ice, while *Askenasia* sp. 6 had the highest read abundance in under-ice water and *Askenasia* sp. 4 in the water column (Table S2 in Online Resource 2).

Different sample types grouped apart also functionally (Fig. 3d, e). Sample type explained 36% and sampling date 21% of the variation in the functions of Ciliophora assemblages (Fig. 3f). Omnivorous Ciliophora predominated in all sample types (Fig. 4b) but less so in sea ice than in under-ice water and the water column—the share of omnivores was on average 85.9%, 97.7% and 96.8% in sea ice, under-ice water and the water column, respectively. In sea ice, cytotrophic (6.5%), bacterivorous (4.9%) and parasitic (2.6%) Ciliophora were the next most abundant feeding types. Sea ice included more surface-oriented Ciliophora (15.8%) than under-ice water (0.8%) or the water column (0.8%), and the latter two were basically planktonic assemblages. Similarly, the share of heterotrophic Ciliophora was higher in sea ice (65.6%) than in under-ice water (53.7%) and the water column (32.4%). Two-thirds of the Ciliophora reads (67.6%) belonged to potentially mixotrophic Ciliophora in the water column.

### H3—Changes in Ciliophora Assemblages in Time

**Fig. 5:**
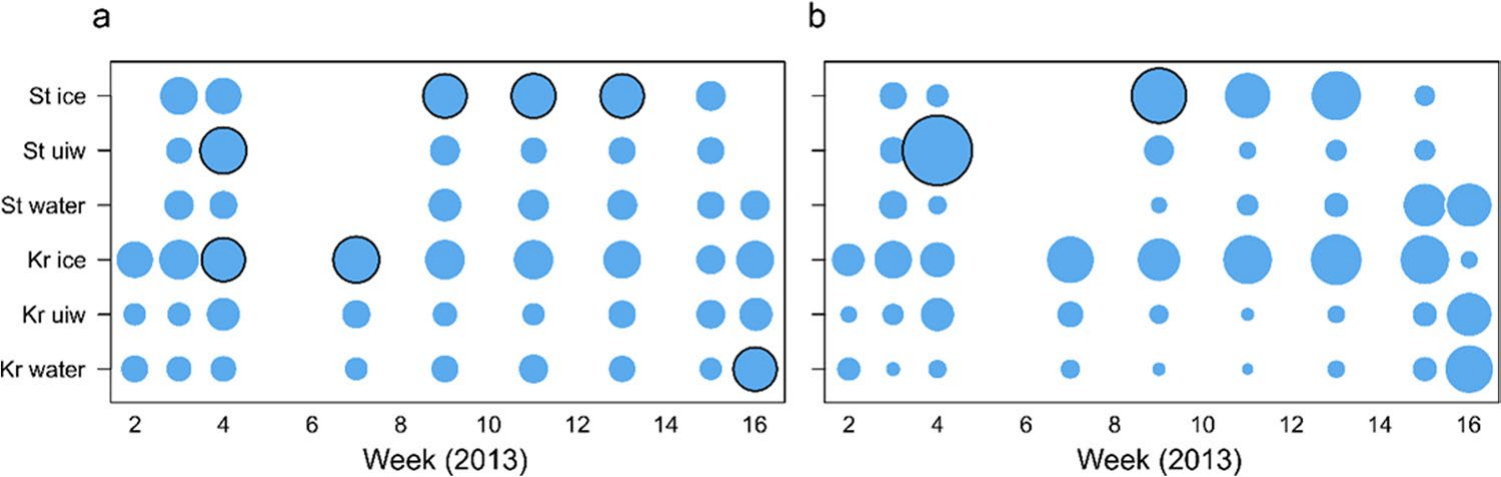
Local contribution to beta diversity (LCBD) values at the different sampling sites (Kr, Krogarviken; St, Storfjärden) and sample types over time of Ciliophora assemblages (**a**) and Ciliophora functions (**b**). The circle surface areas are proportional to the LCBD values. Circles with a black rim indicate significant LCBD values at the 0.05 level. UIW, under-ice water

The share of Strobilidiidae and Strombidiidae of the total Ciliophora reads changed during the winter in under-ice water and the water column (Fig. 4). In midwinter between weeks 4 and 9, Strobilidiidae reads were most abundant in the water column in general, while Strombidiidae reads predominated in early winter and in spring. The same OTU, Strobilidiidae sp. 4, was the predominant Strobilidiidae throughout the season, while Strobilidiidae sp. 3 and Strobilidiidae sp. 5 peaked in read abundance in February–March (Table S2 in Online Resource 2). The predominant Strombidiidae OTUs in early winter were Strombidiida sp. 1, Strombidiida sp. 2 and *Strombidium paracapitatum* 1. In spring, Strombidiida sp. 3 reads became abundant in addition. In sea ice, the same pattern was visible but weaker. This was because of a larger share of *Askenasia*, *Urotricha* and unassigned environmental CONThreeP, Peniculida, Scuticociliatia and Hypotrichia reads in sea ice. Sea-ice samples also showed more significantly different beta-diversity values (5/15) than under-ice water (1/15) and water-column (1/16) samples (*p* < 0.05, random permutations, Fig. 5a), indicating more unique Ciliophora assemblages in sea ice than in water.

Functions followed the taxonomic pattern in water, since Strombidiidae were categorized as potentially mixotrophic and Strobilidiidae as heterotrophic. The share of surface-oriented Ciliophora increased in Krogarviken sea ice during winter until the end of the sea-ice season. They did not increase as clearly in the more dynamic Storfjärden sea ice (with an ice-free period in February and a new ice field after that [36]). Potentially mixotrophic Ciliophora reads were most abundant in sea ice at the beginning and end of the sea-ice season. One sea-ice sample and one under-ice water sample also showed significantly different functional beta-diversity values (Fig. 5b).

Despite the successional changes in the Ciliophora assemblages, there was no temporal trend in taxon or functional richness and evenness (*p* > 0.05, random permutations, Fig. S6 in Online Resource 1). Neither did we find any consistent environmental variable explaining the variation in assemblages or their functions in time (Fig. S7 in Online Resource 1). Based on the Mantel correlograms (Fig. S8 in Online Resource 1), the assemblages obtained in adjacent time points were significantly correlated, and therefore the organisms themselves were the most obvious reason for the time-related changes within them.

## Discussion

Here, we used phylogenetic placement to identify Ciliophora reads obtained from wintertime water and sea ice, and assigned functions to the reads based on this taxonomic information. We showed that sea-ice Ciliophora assemblages were poorer in taxonomic and functional richness than under-ice water and water-column assemblages. Ciliophora diversity was stable throughout the ice-covered season both in sea ice and in water, although the assemblages changed during the course of time. Under-ice water and the water column were distinctly predominated by planktonic Choreotrichida and Oligotrichida, which led to significantly lower taxonomic and functional evenness in water than in sea ice. In addition to Choreotrichida and Oligotrichida, Ciliophora assemblages in sea ice included a set of moderately abundant surface-oriented species. Omnivory was by far the most common feeding type but was not as predominant in sea ice as in water. Sea ice included cytotrophic, bacterivorous and parasitic Ciliophora in addition to the predominant omnivorous Ciliophora. Potentially mixotrophic Ciliophora predominated the water column and heterotrophic Ciliophora sea ice.

When studying organisms living in sea ice, their natural habitat needs to be destroyed either by melting the sea ice or by draining the brine from ice. The brine drainage is not sufficient to sample the sea-ice community as organisms may stick to surfaces in the brine channels [50]. Also melting of ice may cause bias in taxonomic composition due to an osmotic shock during melting (rapid decrease in salinity), and it has been suggested that the lysis of cells can be avoided by melting the samples in an osmotic buffer [51]. However, in DNA-based studies the sample filtration catches both cells and free DNA and, in fact, the cells need to be lysed before DNA extraction. Therefore, the sample processing bias may not be as strong for DNA-based studies as it is for morphological studies. In addition, Rintala et al. [52] showed that buffered melting is not suited for the low-salinity Baltic Sea ice, and the samples should be melted directly without buffer. This direct melting provides an additional benefit for DNA-based methods since it excludes the possibility of environmental DNA contamination originating from the addition of the melting buffer.

The phylogenetic placement approach and the EukRef-Ciliophora database [29] provided better classification resolution than previously [35], and we were able to place the most abundant unnamed sea-ice associated Ciliophora OTUs within Strobilidiidae and assign these Ciliophora functionally as heterotrophic omnivores. Similarly, several other previously unnamed OTUs were placed in the genus *Askenasia* within environmental CONThreeP and were therefore assigned as potentially mixotrophic omnivores. However, a large number of our identifications were reliable at the level of family or higher, and we still needed to construct OTUs to be able to distinguish possible different species within these groups. For example, in the case of the most abundant Ciliophora, Strobilidiidae and Strombidiidae, we were able to confidently assign three species of Strobilidiidae (*Rimostrombidium* sp., *Rimostrombidium veniliae* and *Strobilidium caudatum*) and one Strombidiidae (*Strombidium paracapitatum*, Table S2 in Online Resource 2) out of the 26 and 33 OTUs, respectively. This is because 18S rRNA gene sequences do not allow fine resolution identification, i.e. the gene often has too little variation among the species, and also because we are still missing species from the references [29]. The 18S rRNA gene copy variability can cause additional uncertainty in the identification of the reads as rare variants may be missing from the references, leading to spurious OTUs that in fact are just 18S rRNA gene copy variants of a more abundant OTU [26]. Despite these shortcomings, we reached our goal of better identification resolution than previously and were able to show differences in the obtained habitats.

The significantly lower Ciliophora richness in sea ice than in under-ice water and the water column is because the number of Ciliophora OTUs found only in sea ice (9 OTUs with 1–20 reads; see Table S2 in Online Resource 2) was smaller than the number of Ciliophora OTUs found only in under-ice water and the water column (72 OTUs with 1–914 reads). There are at least two factors that may affect the outcome: the number of sequenced Ciliophora reads across the habitats and the lower Ciliophora diversity in sea ice than in water. One of the most fundamental patterns in ecology is the species abundance distribution, which has a general form of few abundant species and many rare ones occurring within a community, usually described using log-series, Poisson lognormal, or negative binomial models [53]. The more Ciliophora reads are sequenced, the more rare Ciliophora OTUs will be found given the species abundance distribution. This is what we found (Fig. 2 and Fig. S2 in Online Resource 1). However, we extracted the Ciliophora data from studies universally targeting all unicellular eukaryotes [11, 35–37], and more reads were sequenced in total in sea-ice samples than under-ice water or water-column samples (Fig. S2 in Online Resource 1). Therefore, it is likely that Ciliophora diversity is lower in sea ice than in water. Although we did not find clear environmental factors driving the significant differences in Ciliophora assemblages in sea ice, under-ice water and the water column from our set of measured variables, the differences demonstrate that the habitat is a major factor determining which species thrive in these environments, especially in sea ice [18, 54].

We consciously used broad functional categories, although Weisse [4] cautions that this may obscure the results. Nevertheless, our sea-ice samples diverged significantly in functions and harboured variable functions more evenly than the water samples—mainly because of more surface-oriented Ciliophora in sea ice than in water. The presence of surface-oriented Ciliophora in sea ice shows that the sea ice resembles to some extent the benthos, as described previously [15–17, 54]. The surfaces within brine channels are covered by biofilms [14, 54], which attract attaching (e.g. members of the order Sessilida), bacterivorous (unassigned environmental CONThreeP), cytotrophic (*Urotricha* and *Chlamydonellopsis*) and predatory (Lacrymariidae) Ciliophora that utilize the biofilms as food-dense habitats [17, 54]. We showed also that the under-ice water harbours taxonomically and functionally distinct Ciliophora assemblages (Figs. 3, 4, 5). This result highlights the fact that sea ice creates a set of variable habitats in which Ciliophora may play important roles. Algal blooms in the Arctic under-ice water have attracted awareness recently [55], and the fast dividing Ciliophora may readily take advantage of these blooms [56]. It is important to note that with the potential loss of sea ice due to changing climate [57], also under-ice water habitats will disappear, and with the loss of habitats and taxonomic diversity, ecosystem functions may reduce [58].

Ciliophora have pivotal roles in aquatic environments [3, 4]. In our data set, omnivorous mixotrophic Ciliophora (Strombidiidae) predominated in water and omnivorous heterotrophic Ciliophora (Strobilidiidae) in sea ice and under-ice water. This may reflect available prey aggregations: mixotrophy may be functionally more advantageous in the water where prey is more scarcely encountered than in sea ice [3]. However, mixotrophic Ciliophora may form dense occurrences in favourable conditions in sea ice and slush as well [11]. As accumulated research shows (reviewed in Stoecker and Lavrentyev [43]), mixotrophy is undoubtedly more common and more important than previously considered in sea-ice covered areas and warrants specific future research as urged recently [43]. Overall, Ciliophora in ice-covered aquatic environments need further research to pinpoint their important role as efficient channels to transfer primary production to higher trophic levels [43, 56].

## Supplementary Information

Below is the link to the electronic supplementary material.

Supplementary file1 (DOCX 937 KB)

Supplementary file2 (XLSX 524 KB)

Supplementary file3 (TXT 71 KB)

## Data Availability

The datasets analysed during the current study are available in the European Nucleotide Archive (ENA) repository: gene accession numbers: FN689869–FN690738 and study accession numbers PRJEB7625, PRJEB21047 and PRJEB25089.
