# Supplementary material for: Taxonomically and Functionally Distinct Ciliophora Assemblages Inhabiting Baltic Sea Ice
Source: Microb Ecol. 2021 Nov 8;84(4):974–84. doi: 10.1007/s00248-021-01915-4 (PMC9747827; doi:10.1007/s00248-021-01915-4)
Supplement: Supplementary file 1 — Supplementary file1 (DOCX 937 KB) [file 248_2021_1915_MOESM1_ESM.docx]

Title: Taxonomically and functionally distinct Ciliophora assemblages inhabiting Baltic sea ice

Journal: Microbial Ecology

Authors: Markus Majaneva^1,2,3,*^, Janne-Markus Rintala^2,3^, Jaanika Blomster^3^

Affiliations:

^1^Norwegian Institute for Nature Research (NINA), Trondheim, Norway

^2^Tvärminne Zoological Station, University of Helsinki, Hanko, Finland

^3^Department of Environmental Sciences, University of Helsinki, Helsinki, Finland

^*^Corresponding author. Email: [markus.majaneva@gmail.com](mailto:markus.majaneva@gmail.com)

Supplementary methods

Sampling

Ice samples were collected, using a motorized CRREL-type ice-coring auger (9 cm internal diameter; Kovacs Enterprises, Indianapolis, IN, USA) [1-3]. Samples 7 and 8 were collected, using a hand ice saw [1]. Sample 3, about 0.25 m^2^ piece of pancake ice, was collected, using a crane and a metal basket [1]. Slush samples at fast ice station were collected with a shovel from three approximately 50-cm×50-cm squares [2]. The complete ice cores were treated as one sample [1] or 2 to 5 entire ice cores were pooled to ensure there was enough melted sea ice to be comparable to the 2-l water samples [3]. The fast, drift and pack ice samples were cut vertically into five pieces, and each sample included pieces from five replicate ice cores [2].

The under-ice water was sampled by submersing 1-l bottles [2] or 2-l bottles [3] in the corer holes. Water-column samples were collected, using 3-m long (at Krogarviken) and 15-m long (at Storfjärden) hose samplers [3].

The ice core samples (samples 4–6) and the pancake ice sample (sample 3) were left to melt in darkness while floating in 0.2-μm-filtered seawater (salinity 6) at +4°C [1]. The rest ice and slush samples were melted without filtered seawater, following Rintala et al. [4]. To avoid contamination, all sampling equipment used in this study was rinsed with Milli-Q® water prior to sampling. All containers used for melting the ice were washed with a computer-programmed laboratory dishwasher (Deco 260, Dekomed Limited, Cheshire, United Kingdom), using base and acid detergents with five rinsing steps in between and after the washing steps. The containers were then heat sterilized at >85°C for 10 min.

DNA extraction

For the DNA extraction, 500–1000 ml of water, melted sea ice or slush was sequentially filtered with 47-mm-diameter 180-μm pore-size nylon filters (Merck Millipore, Billerica, MA, USA), 20-μm polyvinylidene fluoride filters (Durapore©, Millipore), and 0.2-μm mixed-cellulose ester membrane filters (Schleicher and Schuell Bioscience GmbH, Dassel, Germany). A few exceptions to this general filtration method included samples 1-6, which were filtered with 0.65-μm polyvinylidene fluoride filters (Durapore©, Millipore), and samples 7-8, which were pre-filtered with 40-µm polyvinylidene fluoride filters (Durapore©, Millipore). DNA in studies [1,2] was extracted from the 0.2-µm or 0.65-µm filters, using the phenol–chloroform method of Maggs and Ward [5], and in study [3], using a PowerSoil® DNA Isolation Kit (MO BIO Laboratories).

PCR and sequencing

Amplification of the approximately 1500 bases long fragments of the 18S rRNA gene in Majaneva et al. [1] was done, using the primers UNI7F and UNI1534R [6] and SSUF and SSUR [1] as described in Majaneva et al. [1]. Ten PCR reactions per sample were pooled and the 18S rRNA genes cloned, using the TOPO® Cloning Kit for Sequencing (Invitrogen). The positive colonies were PCR-amplified as described in Majaneva et al. [1]. Cycle sequencing (Sanger sequencing) of the positive clones was carried out, using the Big Dye™ terminator (Applied Biosystems, Foster City, CA, USA) cycling conditions. The samples were loaded on an automated sequencer 3730xl (Applied Biosystems).

Amplification of the approximately 480 bases long 18S rRNA gene fragments (including the variable sites V7, V8 and V9) was done, using primers 18S-F1289 and 18S-R1772 [7], as described in Majaneva et al. [8]. The PCR products were mixed in equimolar ratios and a GS FLX Titanium Rapid Library Preparation Kit (Hoffmann-La Roche, Basel, Switzerland) was used to prepare a DNA library. These pooled libraries were then amplified with beads by emulsion PCR, and the amplified fragments in the DNA libraries were pyrosequenced on a picotitre plate with the 454 GS FLX Titanium system and reagents (Hoffmann-La Roche).

Amplification of the approximately 310 bases long fragment of the V4 region of the 18S rRNA gene was done, using forward primer E572F [9] and reverse primer 897R [10] as described in [3]. Amplification of the approximately 540 bases long fragment of the V4 region was done, using 574*F and 1132R [10] primers as described in Enberg et al. [3] and Majaneva et al. [11]. The amplicons were paired-end sequenced on an Illumina MiSeq instrument using a v3 600-cycle kit (Illumina).

Initial bioinformatic analyses

The chromatograms of the Sanger-sequenced reads were visually inspected, using Chromas Lite 2.1 (Technelysium Pty Ltd), and good-quality reads were assembled using BioEdit software [12]. Taxonomic identification was performed at the National Centre for Biotechnology Information (NCBI) using the Basic Local Alignment Search Tool (BLAST) network service [13].

The 454-sequenced reads were processed in accordance with the QIIME Denoiser UCHIME pipeline described in Majaneva et al. [8], using QIIME 1.8.0 [14] and following the 454 Overview Tutorial and Analysis of the 18S data available in http://qiime.org/tutorials/index.html# (accessed January–March, 2014). Reads with more than six homopolymers, with ambiguous bases, and with greater than zero mismatch in the barcode and primer sequence were removed. Denoiser [15] was used to reduce the sequencing error rate. Chimeric reads were identified, using UCHIME [16]. Taxonomic assignment of the 97% OTUs was done, using SILVA database release 111 [17] within the QIIME program package with UCLUST and the BLAST [13]. If UCLUST failed to assign the OTU, BLAST was used.

The paired-end sequenced MiSeq reads were processed as described in Majaneva et al. [11], using vsearch v2.6.2 [18]. The quality filtering kept 320-380 and 550-610 bases long reads, discarded reads with ambiguous bases and reads with over one maximum expected errors. Chimeric reads were searched, using the command -uchime_denovo in vsearch. OTUs were searched using option fastidious in swarm v2.1.6 [19]. The identity of the OTUs was searched, using the PR2 reference library [20] and using blastn search in BLAST [13] against a nucleotide database at the NCBI. Taxonomy was assigned based on an agreement between the two searches.

Supplementary tables

Table S1. Basic sample information. Sequencing refers to the three different sequencing techniques used here. Sanger refers to Sanger sequencing, 454 refers to Roche 454 sequencing, and MiSeq refers to Illumina MiSeq sequencing. Sample refers to sampling location in the figure S1. Location is given to nearshore sampling stations. UIW = under-ice water, int. = intermediate, Tv = Tvärminne.

| Sequencing | Sample | Material | Date | Area | Location | Reference |
| --- | --- | --- | --- | --- | --- | --- |
| Sanger | 1 | Water | 3. Feb 2006 | Gulf of Finland | Tv Storfjärden | [1] |
| Sanger | 2 | Water | 21. Feb 2006 | Gulf of Finland | Tv Storfjärden | [1] |
| Sanger | 3 | Pancake ice | 3. March 2006 | Bothnian Sea | Bothnian Sea | [1] |
| Sanger | 4 | Ice | 7. Feb 2006 | Gulf of Finland | Tv Storfjärden | [1] |
| Sanger | 5 | Ice | 4. March 2006 | Bothnian Bay | Bothnian Bay | [1] |
| Sanger | 6 | Ice | 21. March 2006 | Gulf of Finland | Tv Storfjärden | [1] |
| Sanger | 7 | Ice | 22. March 2007 | Gulf of Finland | Vuosaari | [1] |
| Sanger | 8 | Ice | 22. March 2007 | Gulf of Finland | Vuosaari | [1] |
| 454 | Drift ice | Surface ice | 9. March 2010 | Gulf of Finland | Gulf of Finland | [2] |
| 454 | Drift ice | Upper int. ice | 9. March 2010 | Gulf of Finland | Gulf of Finland | [2] |
| 454 | Drift ice | Middle ice | 9. March 2010 | Gulf of Finland | Gulf of Finland | [2] |
| 454 | Drift ice | Lower int. ice | 9. March 2010 | Gulf of Finland | Gulf of Finland | [2] |
| 454 | Drift ice | Bottom ice | 9. March 2010 | Gulf of Finland | Gulf of Finland | [2] |
| 454 | Drift ice | UIW | 9. March 2010 | Gulf of Finland | Gulf of Finland | [2] |
| 454 | Pack ice | Surface ice | 11. March 2010 | Gulf of Finland | Gulf of Finland | [2] |
| 454 | Pack ice | Upper int. ice | 11. March 2010 | Gulf of Finland | Gulf of Finland | [2] |
| 454 | Pack ice | Middle ice | 11. March 2010 | Gulf of Finland | Gulf of Finland | [2] |
| 454 | Pack ice | Lower int. ice | 11. March 2010 | Gulf of Finland | Gulf of Finland | [2] |
| 454 | Pack ice | Bottom ice | 11. March 2010 | Gulf of Finland | Gulf of Finland | [2] |
| 454 | Fast ice | Surface ice | 13. March 2010 | Gulf of Finland | Gulf of Finland | [2] |
| 454 | Fast ice | Upper int. ice | 13. March 2010 | Gulf of Finland | Gulf of Finland | [2] |
| 454 | Fast ice | Middle ice | 13. March 2010 | Gulf of Finland | Gulf of Finland | [2] |
| 454 | Fast ice | Lower int. ice | 13. March 2010 | Gulf of Finland | Gulf of Finland | [2] |
| 454 | Fast ice | Bottom ice | 13. March 2010 | Gulf of Finland | Gulf of Finland | [2] |
| 454 | Fast ice | Slush | 13. March 2010 | Gulf of Finland | Gulf of Finland | [2] |
| 454 | Fast ice | Slush | 13. March 2010 | Gulf of Finland | Gulf of Finland | [2] |
| 454 | Fast ice | Slush | 13. March 2010 | Gulf of Finland | Gulf of Finland | [2] |
| 454 | Fast ice | UIW | 13. March 2010 | Gulf of Finland | Gulf of Finland | [2] |
| MiSeq | Time series | Ice | 7. Jan 2013 | Gulf of Finland | Tv Krogarviken | [3,11] |
| MiSeq | Time series | Ice | 14. Jan 2013 | Gulf of Finland | Tv Krogarviken | [3,11] |
| MiSeq | Time series | Ice | 21. Jan 2013 | Gulf of Finland | Tv Krogarviken | [3,11] |
| MiSeq | Time series | Ice | 11. Feb 2013 | Gulf of Finland | Tv Krogarviken | [3,11] |
| MiSeq | Time series | Ice | 27. Feb 2013 | Gulf of Finland | Tv Krogarviken | [3,11] |
| MiSeq | Time series | Ice | 11. March 2013 | Gulf of Finland | Tv Krogarviken | [3,11] |
| MiSeq | Time series | Ice | 25. March 2013 | Gulf of Finland | Tv Krogarviken | [3,11] |
| MiSeq | Time series | Ice | 8. April 2013 | Gulf of Finland | Tv Krogarviken | [3,11] |
| MiSeq | Time series | Ice | 15. April 2013 | Gulf of Finland | Tv Krogarviken | [3,11] |
| MiSeq | Time series | UIW | 7. Jan 2013 | Gulf of Finland | Tv Krogarviken | [3,11] |
| MiSeq | Time series | UIW | 14. Jan 2013 | Gulf of Finland | Tv Krogarviken | [3,11] |
| MiSeq | Time series | UIW | 21. Jan 2013 | Gulf of Finland | Tv Krogarviken | [3,11] |
| MiSeq | Time series | UIW | 11. Feb 2013 | Gulf of Finland | Tv Krogarviken | [3,11] |
| MiSeq | Time series | UIW | 27. Feb 2013 | Gulf of Finland | Tv Krogarviken | [3,11] |
| MiSeq | Time series | UIW | 11. March 2013 | Gulf of Finland | Tv Krogarviken | [3,11] |
| MiSeq | Time series | UIW | 25. March 2013 | Gulf of Finland | Tv Krogarviken | [3,11] |
| MiSeq | Time series | UIW | 8. April 2013 | Gulf of Finland | Tv Krogarviken | [3,11] |
| MiSeq | Time series | UIW | 15. April 2013 | Gulf of Finland | Tv Krogarviken | [3,11] |
| MiSeq | Time series | Water | 7. Jan 2013 | Gulf of Finland | Tv Krogarviken | [3,11] |
| MiSeq | Time series | Water | 14. Jan 2013 | Gulf of Finland | Tv Krogarviken | [3,11] |
| MiSeq | Time series | Water | 21. Jan 2013 | Gulf of Finland | Tv Krogarviken | [3,11] |
| MiSeq | Time series | Water | 11. Feb 2013 | Gulf of Finland | Tv Krogarviken | [3,11] |
| MiSeq | Time series | Water | 27. Feb 2013 | Gulf of Finland | Tv Krogarviken | [3,11] |
| MiSeq | Time series | Water | 11. March 2013 | Gulf of Finland | Tv Krogarviken | [3,11] |
| MiSeq | Time series | Water | 25. March 2013 | Gulf of Finland | Tv Krogarviken | [3,11] |
| MiSeq | Time series | Water | 8. April 2013 | Gulf of Finland | Tv Krogarviken | [3,11] |
| MiSeq | Time series | Water | 15. April 2013 | Gulf of Finland | Tv Krogarviken | [3,11] |
| MiSeq | Time series | Ice | 14. Jan 2013 | Gulf of Finland | Tv Storfjärden | [3,11] |
| MiSeq | Time series | Ice | 21. Jan 2013 | Gulf of Finland | Tv Storfjärden | [3,11] |
| MiSeq | Time series | Ice | 27. Feb 2013 | Gulf of Finland | Tv Storfjärden | [3,11] |
| MiSeq | Time series | Ice | 11. March 2013 | Gulf of Finland | Tv Storfjärden | [3,11] |
| MiSeq | Time series | Ice | 25. March 2013 | Gulf of Finland | Tv Storfjärden | [3,11] |
| MiSeq | Time series | Ice | 8. April 2013 | Gulf of Finland | Tv Storfjärden | [3,11] |
| MiSeq | Time series | UIW | 14. Jan 2013 | Gulf of Finland | Tv Storfjärden | [3,11] |
| MiSeq | Time series | UIW | 21. Jan 2013 | Gulf of Finland | Tv Storfjärden | [3,11] |
| MiSeq | Time series | UIW | 27. Feb 2013 | Gulf of Finland | Tv Storfjärden | [3,11] |
| MiSeq | Time series | UIW | 11. March 2013 | Gulf of Finland | Tv Storfjärden | [3,11] |
| MiSeq | Time series | UIW | 25. March 2013 | Gulf of Finland | Tv Storfjärden | [3,11] |
| MiSeq | Time series | UIW | 8. April 2013 | Gulf of Finland | Tv Storfjärden | [3,11] |
| MiSeq | Time series | Water | 14. Jan 2013 | Gulf of Finland | Tv Storfjärden | [3,11] |
| MiSeq | Time series | Water | 21. Jan 2013 | Gulf of Finland | Tv Storfjärden | [3,11] |
| MiSeq | Time series | Water | 27. Feb 2013 | Gulf of Finland | Tv Storfjärden | [3,11] |
| MiSeq | Time series | Water | 11. March 2013 | Gulf of Finland | Tv Storfjärden | [3,11] |
| MiSeq | Time series | Water | 25. March 2013 | Gulf of Finland | Tv Storfjärden | [3,11] |
| MiSeq | Time series | Water | 8. April 2013 | Gulf of Finland | Tv Storfjärden | [3,11] |
| MiSeq | Time series | Water | 15. April 2013 | Gulf of Finland | Tv Storfjärden | [3,11] |

Supplementary figures


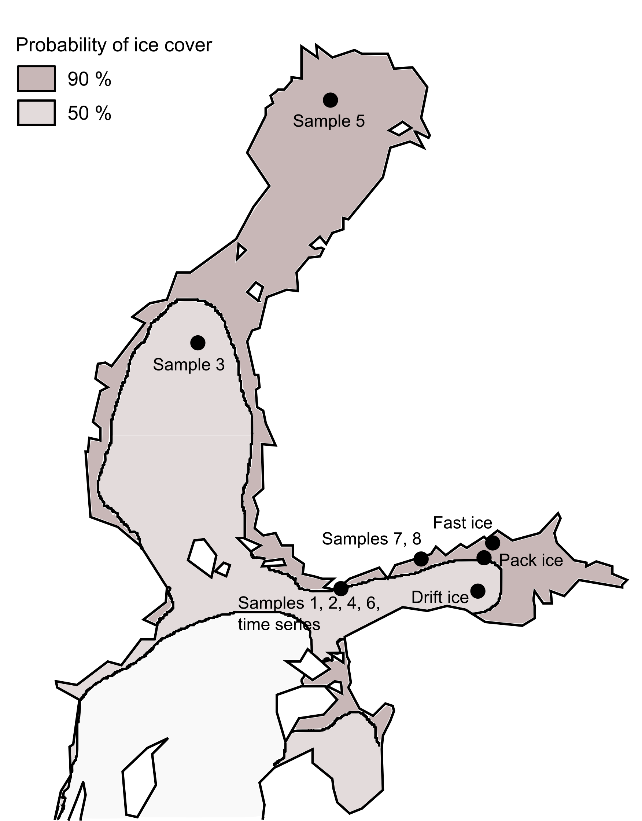


Fig. S1. Map of the northern Baltic Sea. The probability of ice cover is redrawn from Thomas et al. [21], Fig. 9.2, showing the areas with the highest probabilities of ice cover. The sampling sites are redrawn from Majaneva [22]. The numerical samples are the Sanger-sequencing samples from Majaneva et al. [1], fast, pack and drift ice sites are the 454 Roche-sequenced samples from Majaneva et al. [2], and the time series is the Illumina MiSeq samples from Enberg et al. [3]. Sea-ice and under-ice water samples were collected offshore from the Gulfs of Bothnia and Finland during two sea-ice cruises: onboard RV *Maria S. Merian* (3–4 March 2006, Majaneva et al. [1]) and RV *Aranda* (8–19 March 2010, Majaneva et al. [2]). Coastal sea-ice and under-ice water samples were collected from the vicinity of the Tvärminne Zoological Station (7 February and 21 March 2006, Majaneva et al. [1], and biweekly between 7 January and 22 April 2013, [3]) and the Vuosaari harbour, Helsinki (22 March 2007, [1]).


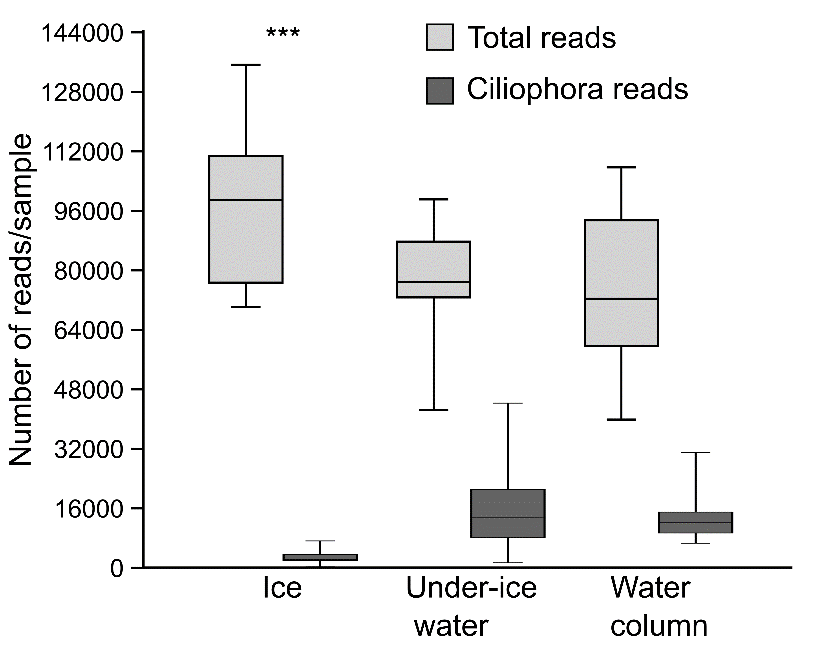


Fig. S2. Number of total and Ciliophora reads in the time-series samples. Sea-ice samples (***, 15 samples) had significantly higher number of total reads but significantly lower number of Ciliophora reads than under-ice water (15 samples) and water-column samples (16 samples) according to one-way analysis of variance (ANOVA) (total reads: F = 7.74, p = 0.001; Ciliophora reads: F = 15.16, p = 1.04E-05) and following Tukey’s pairwise comparisons (total reads: ice – under-ice water Tukey’s Q = 4.50, p = 0.008; ice – water column: Q = 5.11, p = 0.002; Ciliophora reads: ice – under-ice water Tukey’s Q = 7.37, p = 0.0001; ice – water column: Q = 6.03, p = 0.0004).


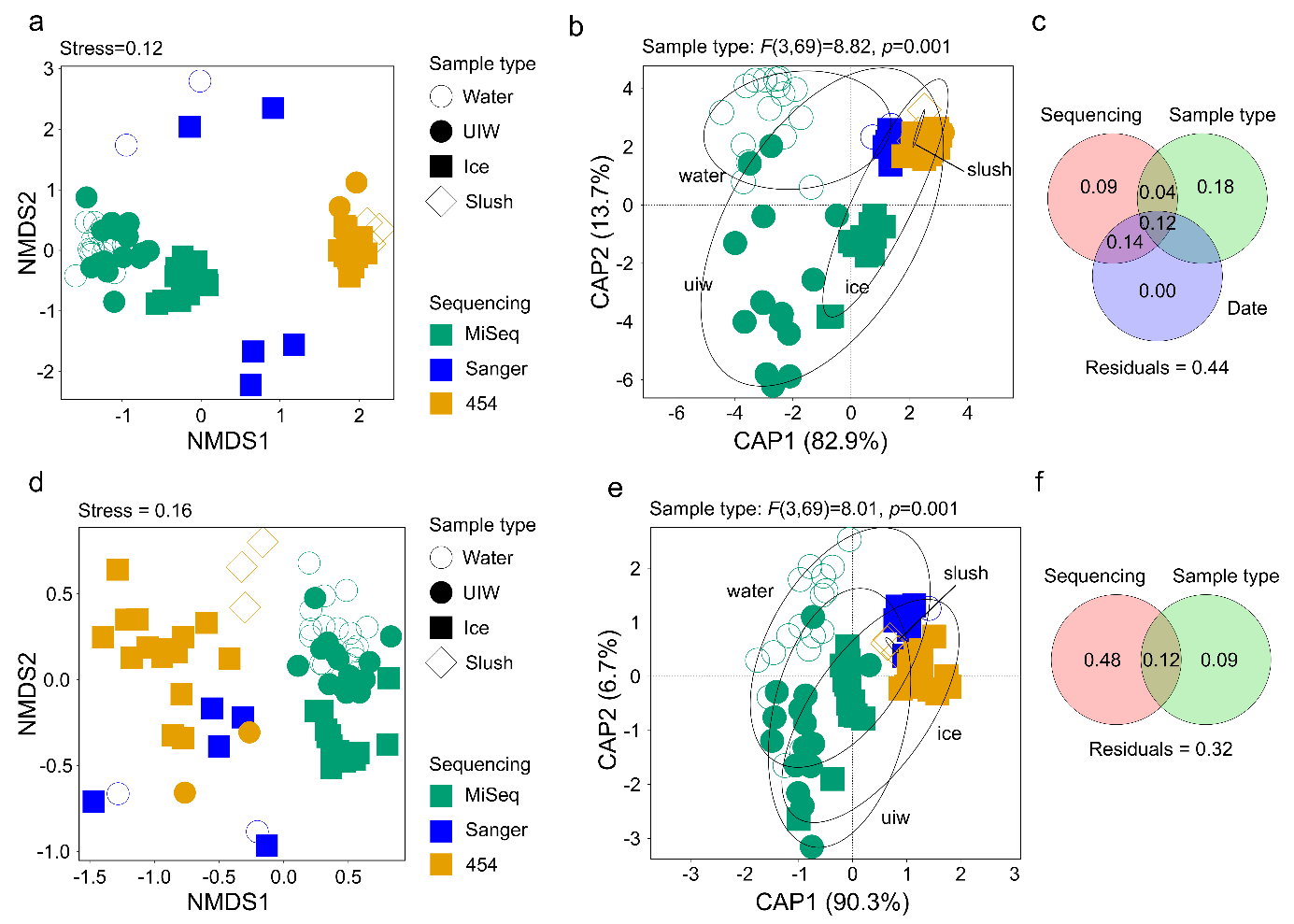


Fig. S3. Differences in Ciliophora assemblages and their functions. (a, d) non-metric multidimensional scaling (NMDS) plots based on Bray–Curtis dissimilarity indices of the Ciliophora assemblages in the different samples. (a) Taxonomic composition, (d), functional composition. (b, e) Distance-based redundancy analysis plots based on binomial distribution of the Ciliophora assemblages in the different samples. Significance was tested with a permutation test (999 permutations, significance level p < 0.05) and following pairwise Adonis with Holm-corrected p-values. (b) Taxonomic composition. Ciliophora assemblages in sea-ice samples were significantly different than in under-ice water and water-column samples (p = 0.006), assemblages in slush were significantly different than in the water-column (p = 0.012) and under-ice water samples (p = 0.016), and assemblages in under-ice water differed significantly from those in the water column (p = 0.012). (e) Functional composition. Functions in ice differed significantly from functions in water and under-ice water (p = 0.006), and functions in slush differed significantly from functions in under-ice water (p = 0.040) and in the water column (p = 0.048). (c, f) Venn diagrams showing variation partitioning for Ciliophora assemblages.


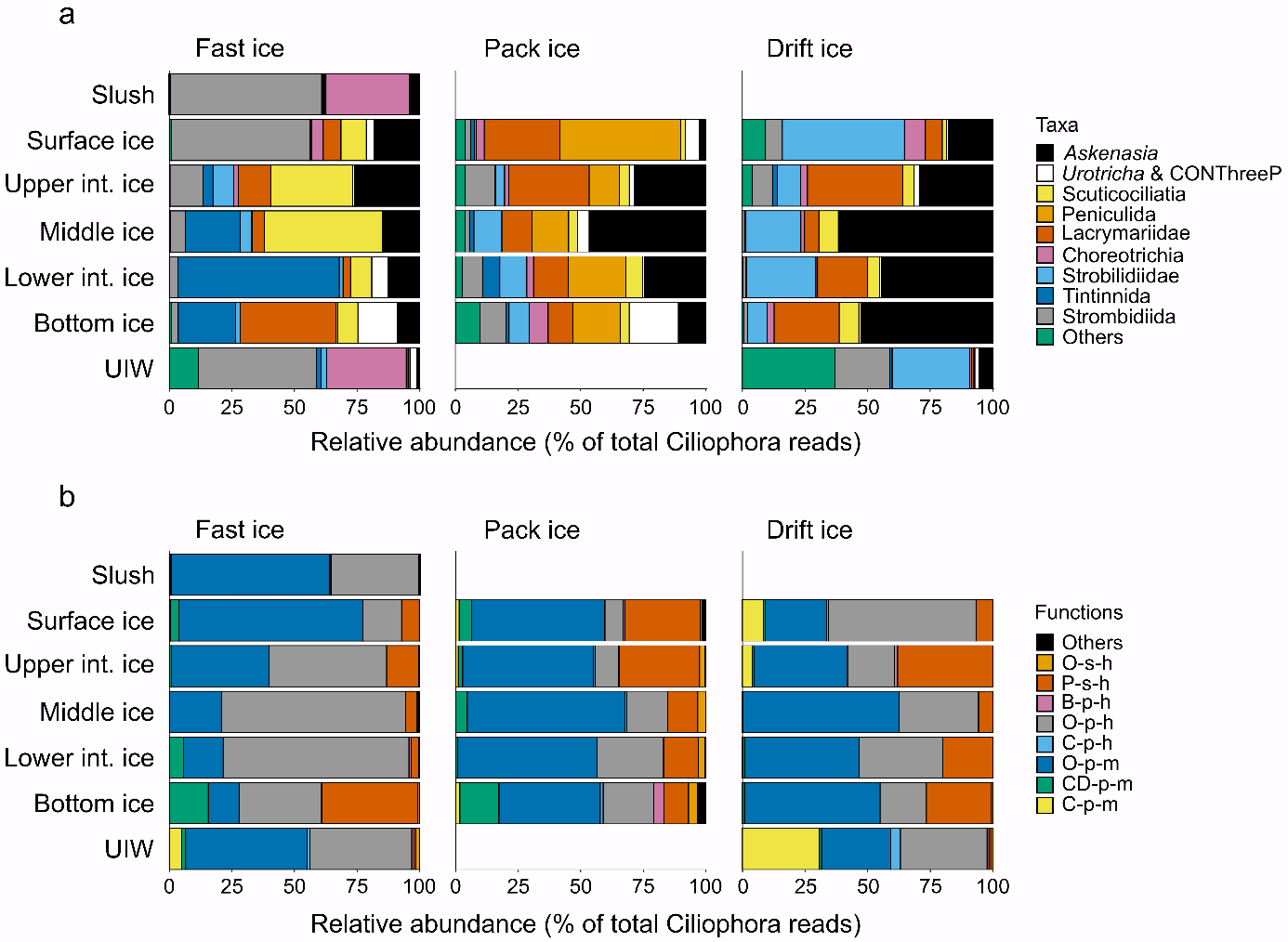


Fig. S4. Vertical distribution of Ciliophora assemblages. (a) Relative abundance of 98% Ciliophora operational taxonomic units (OTUs) in different vertical layers of sea ice at three different sea-ice type stations. (b) Relative abundance of Ciliophora functional groups in different vertical layers of sea ice at three different sea-ice type stations. The abbreviated functions include: (B) bacteria filtration, (O) omnivorous, (C) cytrophic, (P) predatory, (D) occasionally detritivorous, (s) surface dwelling, (p) planktonic, (h) heterotrophic and (m) potentially mixotrophic. OTUs and functions were merged to show the most abundant groupings. UIW = under-ice water. int. = intermediate.


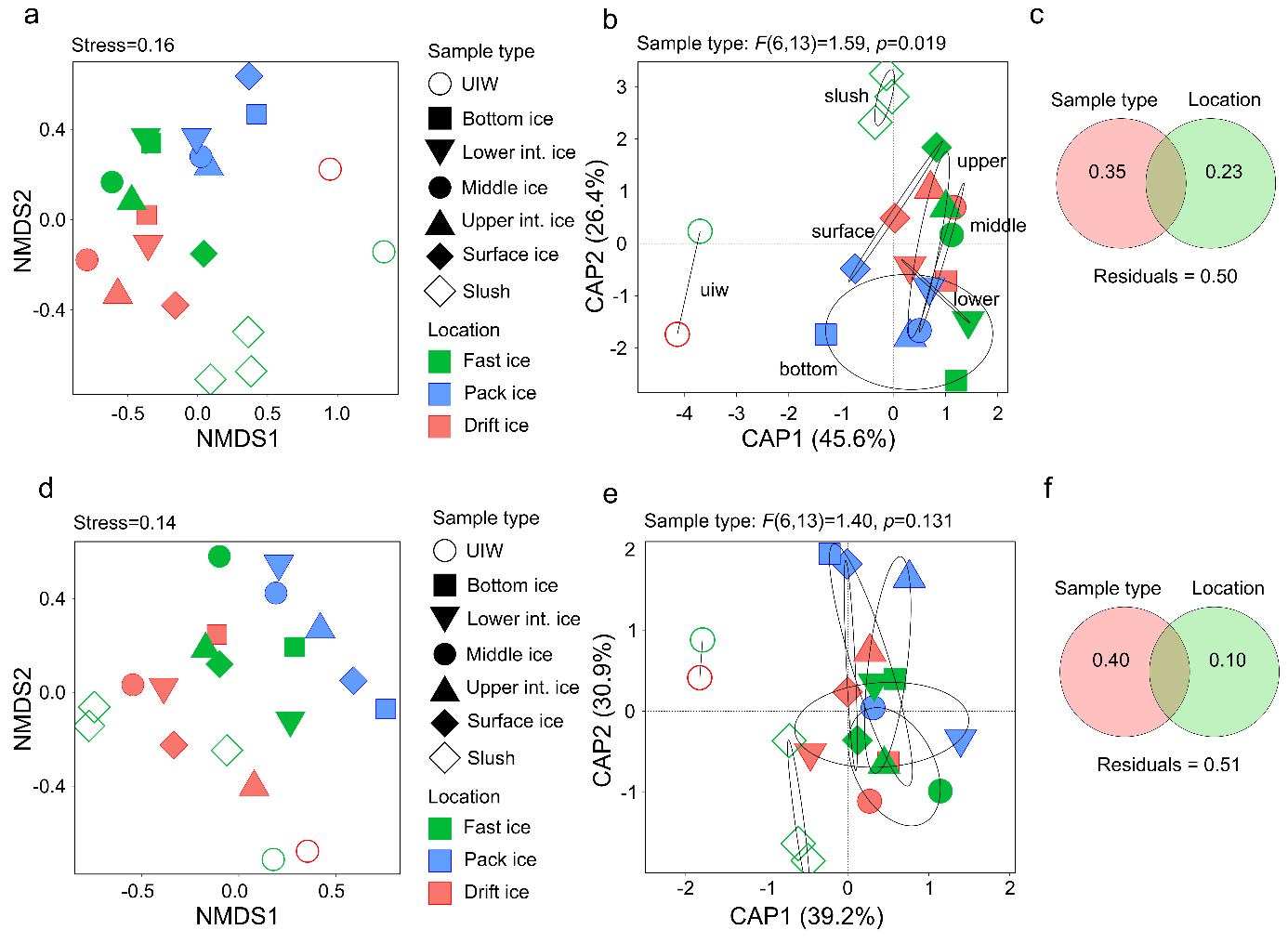


Fig. S5. Differences in Ciliophora assemblages and their functions. (a, d) non-metric multidimensional scaling (NMDS) plots based on Bray–Curtis dissimilarity indices of the Ciliophora assemblages in the different samples. (a) Taxonomic composition, (d), functional composition. (b, e) Distance-based redundancy analysis plots based on binomial distribution of the Ciliophora assemblages in the different samples. Significance was tested with a permutation test (999 permutations, significance level p < 0.05) and following pairwise Adonis with Holm-corrected p-values. (b) Taxonomic composition. Ciliophora assemblages in sea ice differed significantly from slush (*p* = 0.026) and under-ice water (*p* = 0.015) samples. (e) Functional composition. No differences in functions were found. (c, f) Venn diagrams showing variation partitioning for Ciliophora assemblages.


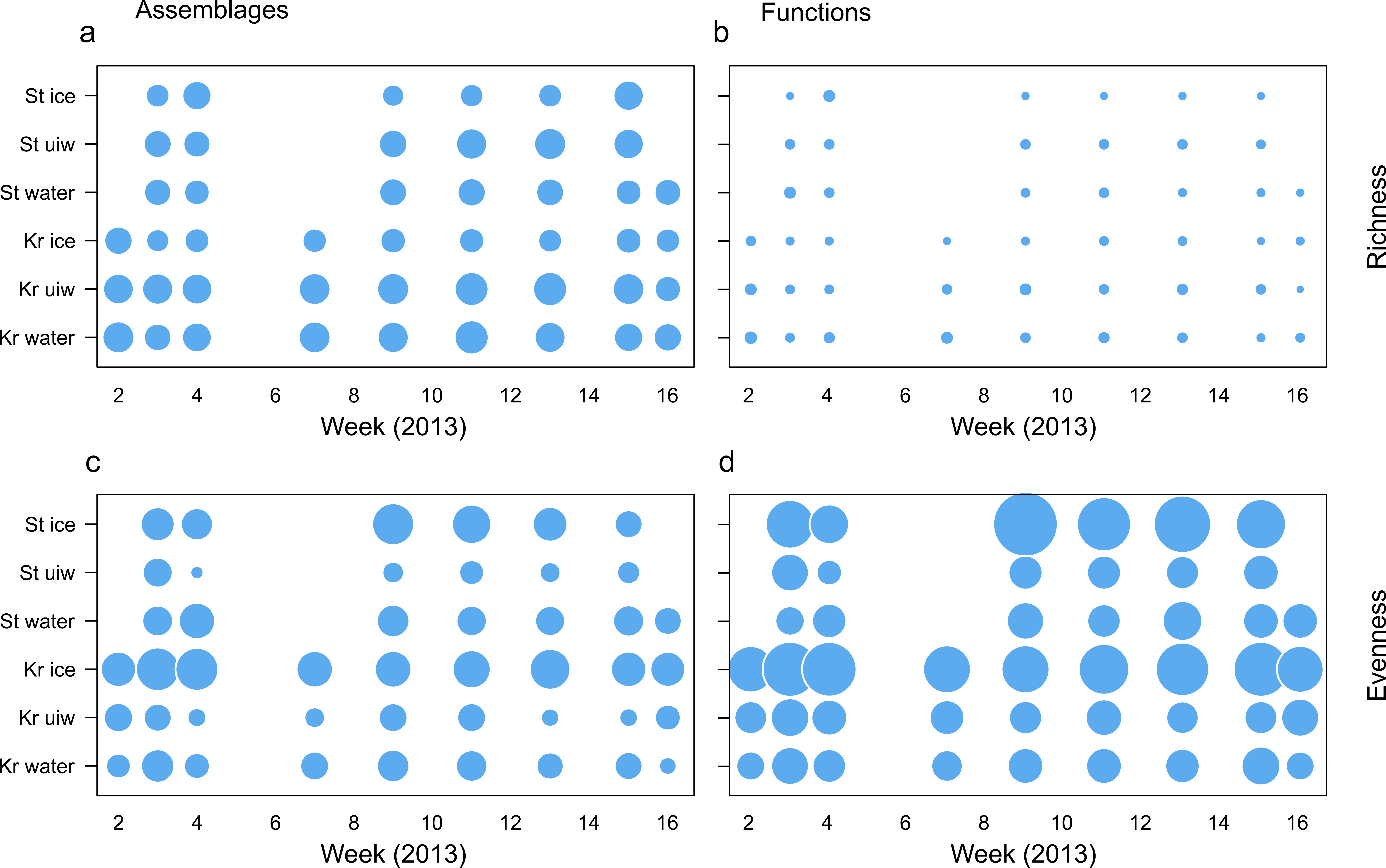


Fig. S6. Space–time maps of taxonomic richness (a), functional richness (b), taxonomic evenness (c) and functional evenness (d). Within each map, the surface area of the circles is proportional to index values. Kr = Krogarviken, St = Storfjärden, UIW = under-ice water.


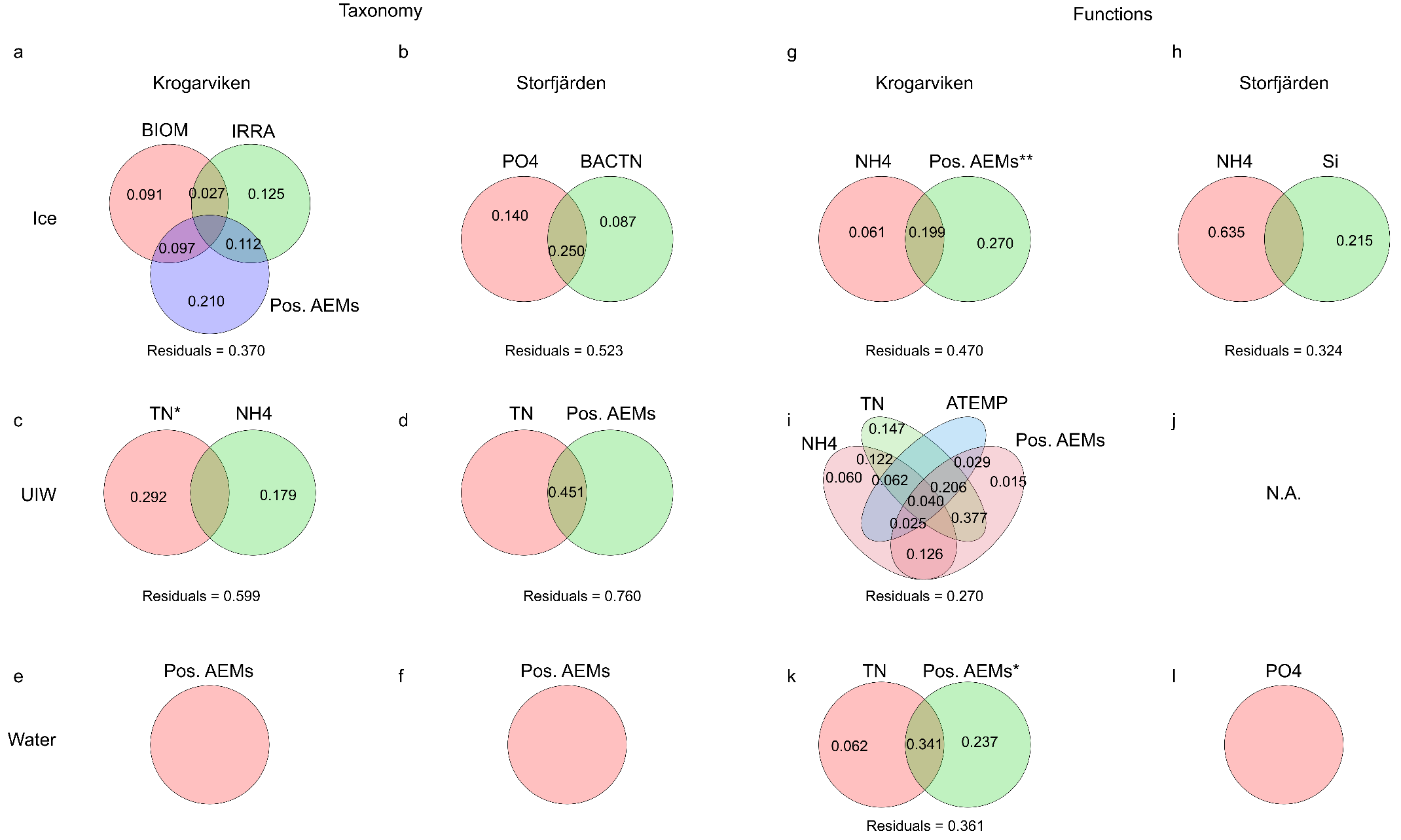


Fig. S7. Venn diagrams illustrating the relative contribution of environmental and asymmetric eigenvector map (AEM) explanatory variables with positive Moran I to taxonomic (a–f) and functional (g–l) Ciliophora diversity. The variables that were forward selected (alpha=10, 9999 permutations, *p* ≤ 0.08) included algal biomass (BIOM), bacterial abundance (BACTN), daily irradiance (IRRA), air temperature (ATEMP), phosphate (PO4), total nitrogen (TN), ammonium (NH4), silicate (Si) and positive AEMs (Pos. AEMs). The fractions of variations represent the adjusted R^2^ values. Negative fraction values are not presented. ** indicates significant partial contribution of variables for *p* < 0.001 and * indicates significant partial contribution of variables for *p* < 0.05 using a permutation test for redundancy analysis (999 permutations). (a) Krogarviken ice, (b) Storfjärden ice, (c) Krogarviken under-ice water, (d) Storfjärden under-ice water, (e) Krogarviken water-column and (f) Storfjärden water-column samples. (g) Krogarviken ice, (h) Storfjärden ice, (i) Krogarviken under-ice water, (j) Storfjärden under-ice water (did not include any), (k) Krogarviken water-column and (l) Storfjärden water-column samples. N.A. = not applicable, UIW = under-ice water.


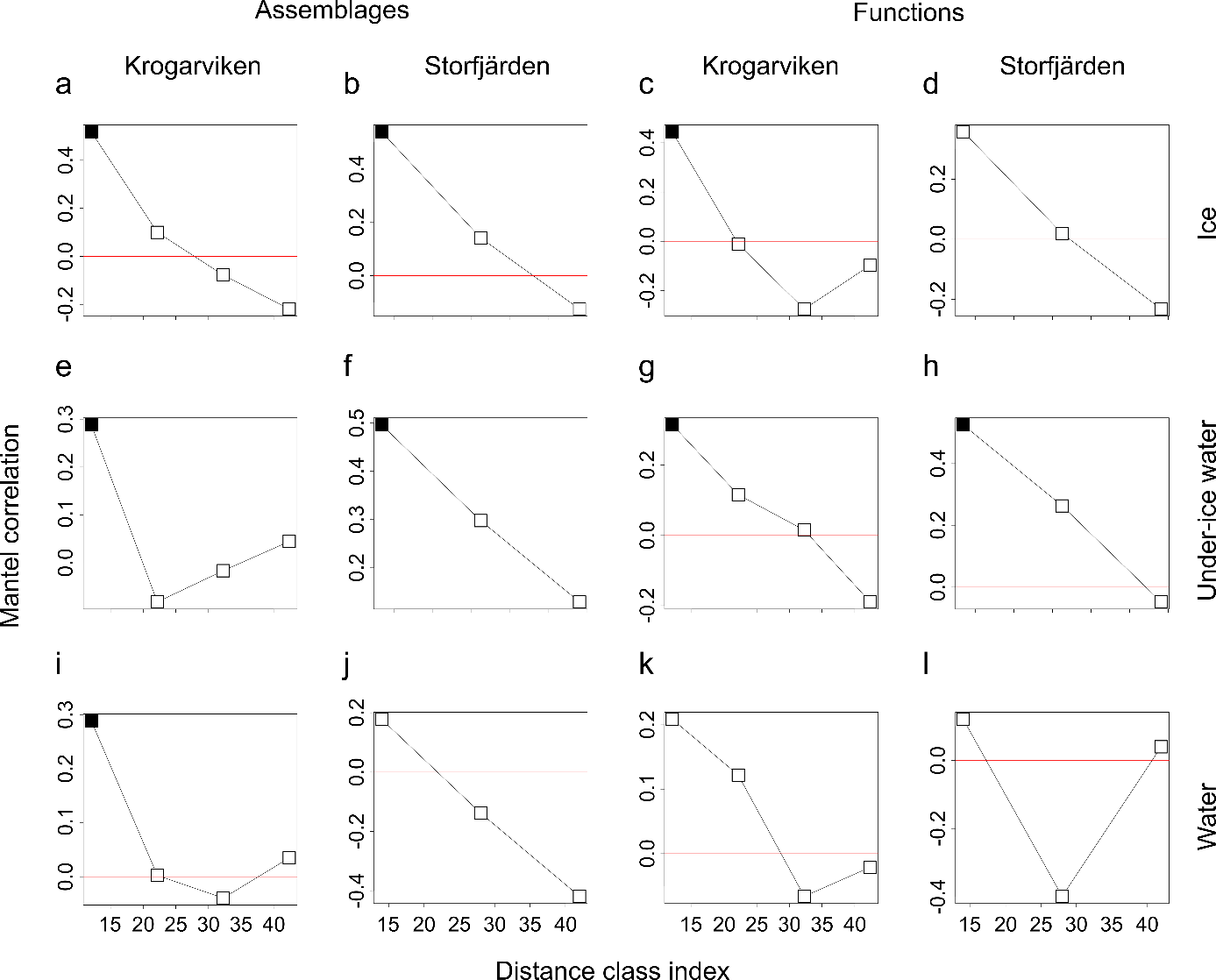


Fig. S8. Multivariate Mantel correlograms of the sites. Significant correlations are marked with black squares. Hellinger-transformed Ciliophora data (a, b, e, f, i, j) and Hellinger-transformed functions data (c, d, g, h, k, l) from Krogarviken (a, e, i, c, g, k) and Storfjärden (b, f, j, d, h, l) and from ice (a, b, c, d), under-ice water (e, f, g, h) and the water column (i, j, k, l). Maximum and significant positive correlations are found in the first distance class, which corresponds to pairs of observations seven days apart. Larger distance classes show no significant temporal correlation.

Supplementary references

1. Majaneva M, Rintala J-M, Piisilä M, Fewer DP, Blomster J (2012) Comparison of wintertime eukaryotic community from sea ice and open water in the Baltic Sea, based on sequencing of the 18S rRNA gene. Polar Biology 35:875-889
2. Majaneva M, Blomster J, Müller S, Autio R, Majaneva S, Hyytiäinen K, Nagai S, Rintala J-M (2017) Sea-ice eukaryotes of the Gulf of Finland, Baltic Sea, and evidence for herbivory on weakly shade-adapted ice algae. European journal of protistology 57:1-15
3. Enberg S, Majaneva M, Autio R, Blomster J, Rintala J-M (2018) Phases of microalgal succession in sea ice and the water column in the Baltic Sea from autumn to spring. Marine Ecology Progress Series 599:19-34
4. Rintala J-M, Piiparinen J, Blomster J, Majaneva M, Müller S, Uusikivi J, Autio R (2014) Fast direct melting of brackish sea-ice samples results in biologically more accurate results than slow buffered melting. Polar Biology 37:1811-1822
5. Maggs CA, Ward BA (1996) The genus *Pikea* (Dumontiaceae, Rhodophyta) in England and the North Pacific: comparative morphological, life history, and molecular studies. Journal of Phycology 32:176-193
6. Moon-van der Staay SY, De Wachter R, Vaulot D (2001) Oceanic 18S rDNA sequences from picoplankton reveal unsuspected eukaryotic diversity. Nature 409:607-610
7. Nishitani G, Nagai S, Hayakawa S, Kosaka Y, Sakurada K, Kamiyama T, Gojobori T (2012) Multiple plastids collected by the dinoflagellate *Dinophysis mitra* through kleptoplastidy. Applied and Environmental Microbiology 78:813-821
8. Majaneva M, Hyytiäinen K, Varvio SL, Nagai S, Blomster J (2015) Bioinformatic amplicon read processing strategies strongly affect eukaryotic diversity and the taxonomic composition of communities. PloS ONE 10:e0130035
9. Comeau AM, Li WKW, Tremblay J-E, Carmack EC, Lovejoy C (2011) Arctic Ocean Microbial Community Structure before and after the 2007 Record Sea Ice Minimum. PLoS ONE 6:e27492
10. Hugerth LW, Muller EEL, Hu YOO, Lebrun LAM, Roume H, Lundin D, Wilmes P, Andersson AF (2014) Systematic design of 18S rRNA gene primers for determining

eukaryotic diversity in microbial consortia. PLoS ONE 9:e95567

1. Majaneva M, Enberg S, Autio R, Blomster J, Rintala J-M (2019) Mamiellophyceae shift in seasonal predominance in the Baltic Sea. Aquatic Microbial Ecology 83:181-187
2. Hall TA (1999) Bioedit: a user-friendly biological sequence alignment editor and analysis program for windows 95/98/nt. Nucleic Acids Symposium Series 41:95-98
3. Altschul SF, Madden TL, Schäffer AA, Zhang J, Zhang Z, Miller W, Lipman DJ (1997) Gapped BLAST and PSI-BLAST: a new generation of protein database search programs. Nucleic Acids Research 25:3389-3402
4. Caporaso JG, Kuczynski J, Stombaugh J, Bittinger K, Bushman FD, Costello EK, Fierer N, Gonzalez Peña A, Goodrich JK, Gordon JI, Huttley GA, Kelley ST, Knights D, Koenig JE, Ley RE, Lozupone CA, McDonald D, Muegge BD, Pirrung M, Reeder J, Sevinsky JR, Turnbaugh PJ, Walters WA, Widmann J, Yatsunenko T, Zaneveld J, Knight R (2010) QIIME allows analysis of high-throughput community sequencing data. Nature Methods 7:335-336.
5. Reeder J, Knight R (2010) Rapidly denoising pyrosequencing amplicon reads by exploiting rank-abundance distributions. Nature Methods 7:668-669
6. Edgar RC, Haas BJ, Clemente JC, Quince C, Knight R (2011) UCHIME improves sensitivity and speed of chimera detection. Bioinformatics 27:2194-2200
7. Quast C, Pruesse E, Yilmaz P, Gerken J, Schweer T, Yarza P, Peplies J, Glöckner FO (2013) The SILVA ribosomal RNA gene database project: improved data processing and web-based tools. Nucleic Acids Research 41:D590-D596
8. Rognes T, Flouri T, Nichols B, Quince C, Mahé F (2016) VSEARCH: a versatile open source tool for metagenomics. PeerJ 4:e2584
9. Mahé F, Rognes T, Quince C, de Vargas C, Dunthorn M. (2015) Swarm v2: highly-scalable and high-resolution amplicon clustering. PeerJ 3:e1420
10. Guillou L, Bachar D, Audic S, Bass D, Berney C, Bittner L, Boutte C, Burgaud G, de Vargas C, Decelle J, del Campo J, Dolan JR, Dunthorn M, Edvardsen B, Holzmann M, Kooistra WHCF, Lara E, Le Bescot N, Logares R, Mahe F, Massana R, Montresor M, Morard R, Not F, Pawlowski J, Probert I, Sauvadet A-L, Siano R, Stoeck T, Vaulot D, Zimmermann P, Christen R (2013) The Protist Ribosomal Reference database (PR2): a catalog of unicellular eukaryote Small Sub-Unit rRNA sequences with curated taxonomy. Nucleic Acids Research 41:D597-604
11. Thomas DN, Kaartokallio H, Tedesco L, Majaneva M, Piiparinen J, Eronen-Rasimus E, Rintala J-M, Kuosa H, Blomster J, Vainio J, Granskog MA (2017) Life associated with Baltic Sea ice. In: Snoeijs-Leijonmalm P, Schubert H, Radziejewska T (eds) Biological Oceanography of the Baltic Sea. Springer, Dordrecht, pp 333-357
12. Majaneva M (2013) Linking taxonomy and environmental 18S-rRNA-gene sequencing of Baltic Sea protists. Walter and Andrée de Nottbeck Foundation Scientific Reports 40:1-49
